# Recent Advances in Single-Molecule Tracking and Imaging Techniques

**DOI:** 10.1146/annurev-anchem-091922-073057

**Published:** 2023-06-14

**Authors:** Trung Duc Nguyen, Yuan-I Chen, Limin H. Chen, Hsin-Chih Yeh

**Affiliations:** 1 Department of Biomedical Engineering, University of Texas at Austin, Austin, Texas, USA; 2 Texas Materials Institute, University of Texas at Austin, Austin, Texas, USA

**Keywords:** single-molecule tracking, feedback-control tracking, point-spread-function engineering, multiple plane microscopy, trajectory analysis, clustering analysis

## Abstract

Since the early 1990s, single-molecule detection in solution at room temperature has enabled direct observation of single biomolecules at work in real time and under physiological conditions, providing insights into complex biological systems that the traditional ensemble methods cannot offer. In particular, recent advances in single-molecule tracking techniques allow researchers to follow individual biomolecules in their native environments for a timescale of seconds to minutes, revealing not only the distinct pathways these biomolecules take for downstream signaling but also their roles in supporting life. In this review, we discuss various single-molecule tracking and imaging techniques developed to date, with an emphasis on advanced three-dimensional (3D) tracking systems that not only achieve ultrahigh spatiotemporal resolution but also provide sufficient working depths suitable for tracking single molecules in 3D tissue models. We then summarize the observables that can be extracted from the trajectory data. Methods to perform single-molecule clustering analysis and future directions are also discussed.

## 1. INTRODUCTION

Fluorescence microscopy is an essential technique in all biological and biomedical laboratories. Laser-induced fluorescence offers researchers direct visual access to the structures and organizations of the intracellular environment. With the significant progress made in instrumentation and experimental methods in the 1980s and 1990s (1, 2), detection of single fluorophores in solution at room temperature became feasible (3–7), allowing researchers to see stochastic processes or minor reaction pathways embedded in the systems that would otherwise be masked in the conventional ensemble measurements (8–10). In the past three decades, there has been an explosive development in the methods to detect, image, and track single molecules in solution, on surfaces, or in live cells. These methods are collectively termed single-molecule tracking (SMT) in this review, including both single-molecule imaging (SMI) techniques and single-particle tracking (SPT) on fluorescent nanoparticle probes. SMT methods have given researchers unprecedented power to monitor molecular behaviors in live cells, seeing how a molecule jostles around, collides with, binds to, and dissociates from another molecule in its proximity (11–14). Moreover, advanced SMT techniques allow researchers to see a group of molecules concentrate, cluster, organize, and dissociate in a cellular environment (15, 16), not only in the two-dimensional (2D) space (17–20) but also in the three-dimensional (3D) (11, 21–25) space, or to follow an individual single molecule for more than 10 min (26). Although this review aims to provide a broad overview of the topic, considering the enormous diversity of SMT techniques and research, readers are advised to look to previous reviews of this topic and its manifold applications (27–33).

SMT techniques have answered many fundamental questions in biology. These include elucidating the motional mode of myosin V walking on the actin filament (34), revealing the selective cargo transport mechanisms through the nuclear pore complex (19), validating the drug efficacy in blocking glycosylated programmed death-ligand 1 (PD-L1) and inducing its internalization (24), clarifying the search mechanisms that transcription factors take to find their targets (35, 36), demonstrating the compartmentalization of highly dynamic plasma membranes (14), and identifying the agonist-specific dimer formation of opioid receptors (20). Considering the enormous diversity of single-molecule research nowadays, any study aimed at completely reviewing all of SMT research is a futile undertaking. SMT is not only beneficial for following single molecules and seeing their movement patterns and transport routes in live cells, but it can also detect the activation of important signaling molecules, often through fluorescence modulation schemes such as fluorescence resonance energy transfer (FRET). For instance, activation of a single G protein Ras (tagged with an acceptor) can be observed upon its binding with a GTP (guanosine triphosphate) (tagged with an acceptor) on a total internal reflection fluorescence (TIRF) microscope (37). Similarly, transient duplex formation between an oligonucleotide (tagged with a donor) and its complementary strand (tagged with a quencher) can also be observed in live cells using a custom-built confocal-feedback 3D-SMT system (11).

Whereas molecular, cellular, and developmental biology textbooks today are all filled with beautiful schematics showing molecular interactions, complex formations, transport processes, and signaling pathways, most of these schematics fail to provide any information on timescales, rate constants, equilibrium constants, delivery routes, motion speeds, and oligomer sizes of the molecular machine at work (27). In addition, many of the schematics were derived from the conventional biochemical analysis and in vitro binding assays, such as pull-down assays, fluorescence anisotropy, and surface plasmon resonance measurements, making these schematics speculative rather than definitive. For example, many receptors in the plasma membrane repeatedly switch between fast and slow diffusion modes (15, 17, 18, 21, 22), indicating transient interactions with their environment or formation of molecular complexes and oligomers. These transient molecular behaviors could not have been observed by any ensemble measurements but were visualized through SMT techniques. Moreover, modern SMT methods are capable of observing multicolor species (38–40) and provide a temporal resolution down to 33 μs (on tracking a single organic dye) (41) and 10 μs (on tracking a nanocrystal) (42). The capability to see biomolecules or viral particles in action gives SMT a unique position in quantitative biology and analytical chemistry.

Although significant progress has been made in instrumentation, experimental approaches, and trajectory analyses, SMT is still limited by the currently available fluorescent labels. Virtually all fluorescence methods for single-molecule detection and analysis are indirect in that they do not detect the molecule of interest but a fluorescent label (or a number of labels) that tags the molecule. SMT in the early days required single molecules to be heavily labeled (43). But even with improved instrumentation, multiple labeling strategies (44–46) are still used today in SMT for various reasons. Here, we focus on tracking single molecules labeled with only a single fluorescent tag, thus bypassing any unwanted effects from the multiply labeled systems.

New generations of fluorescent tags and labeling techniques have fueled the development of novel SMT techniques, offering researchers unprecedented visual access to the inner workings of the cells. The SMT community is constantly pushing for fluorophores that are smaller, brighter, more photostable, less blinking, and easier in their genetic encoding processes (47). Besides, fluorophores having a palette of colors (48–50) and diverse fluorescence lifetimes (51) are desirable for simultaneous tracking of multiple molecular species (40). Early SMT works relied on genetically encoded green fluorescent protein (GFP) and its relatives, such as PA-GFP, Dendra2, and mEOS3 (52–54), for cellular studies (55, 56). But the low brightness and poor photostability of fluorescent proteins, as compared to organic dyes (54), made some SMT results questionable (47). Although organic dyes derived from rhodamine, coumarin, xanthene, and cyanine [e.g., Alexa, ATTO, and Cy fluorophores (57)] exhibit much-improved photophysical and photochemical properties, conjugating these dyes to the molecules of interest is not straightforward, and electroporation or microinjection of the labeled molecules into cells is often required for SMT experiments (11, 58). Semiconductor nanocrystals (59, 60) and fluorescent beads (21) are still used for SMT, but they are mostly restricted to labeling receptors on the plasma membranes for tracking.

A probe strategy that became popular in recent years is chemigenetic labels (also called hybrid labels) that combine genetically encodable protein tags with cell-permeable dyes for labeling the proteins of interest. For example, the HaloTag protein is a bacterial dehalogenase variant that binds rapidly and specifically to a synthetic ligand of choice (61). Expression of the protein of interest with a HaloTag, followed by labeling the HaloTag with a cell-permeable, ligand-functionalized organic dye, enables reliable and long-term tracking of single molecules inside live cells (26). Other chemigenetic tags, such as SNAP-tag (62) and CLIP-tag (63), are based on similar principles, thus allowing for orthogonal labeling and multicolor imaging (20, 29, 64). Recent advances in cell-permeable dyes (49, 65, 66) have further promoted the use of chemigenetic strategy in SMT. Tetramethylrhodamine (TMR) derivatives such as Janelia Fluor JF_549_ (67, 68) and silicon-containing rhodamine derivatives such as JF_646_ (29) and SaraFluor SF_650_ (69) are frequently used by SMT researchers today, owing to their improved photostability (as compared to TMR) and great labeling efficiency [SNAP-tag can have >90% labeling efficiency (20)]. Interestingly, it was recently found that the HaloTag-JF_549_ outperforms SNAP-tag-JF_549_ or CLIP-tag-JF_549_ in SMT experiments, showing higher photostability and less nonspecific binding (47). It was also found that by creating HaloTag variants (HT7, HT9, HT10 and HT11), HaloTag-TMR complexes can have four different lifetimes, ranging from 1.34 to 2.80 ns (51). The rapid development of chemigenetic labeling techniques will soon allow researchers to differentiate the tracked molecules not only by their fluorescence colors but also by their fluorescence lifetimes. In the following sections, we continue to describe how the recent advances in instrumentation give SMT a deeper, longer, and clearer view of the dynamic processes of the molecules of interest.

## 2. TWO-DIMENSIONAL SINGLE-MOLECULE TRACKING AND IMAGING TECHNIQUES

**Figure 1:**
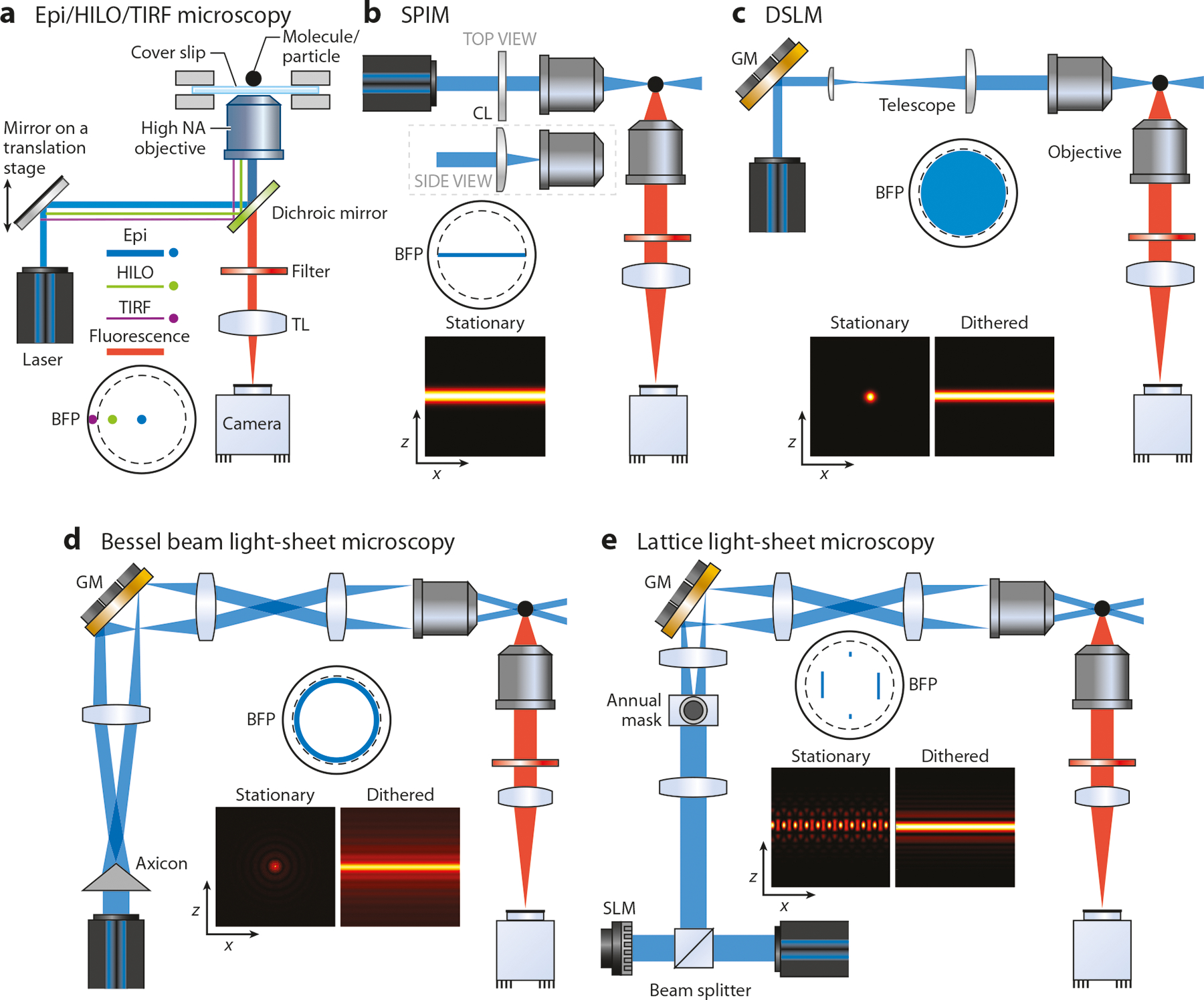
Different 2D-SMT and imaging techniques. (*a*) Epi/HILO/TIRF microscopy (87), HILO (80), and TIRF (88). (*b*) SPIM (83). (*c*) DSLM (89). (*d*) Bessel beam light-sheet microscopy (90). (*e*) Lattice light-sheet microscopy (91) for generating an ultrathin illumination plane and a large field of view. The dashed circle at the BFP denotes the critical angle position (assuming a glass/water interface). Abbreviations: 2D, two-dimensional; BFP, back focal plane; CL, cylindrical lens; DSLM, digital scanned laser light-sheet fluorescence microscopy; Epi, epiluminescence; GM, galvo mirror; HILO, highly inclined and laminated optical sheet; NA, numerical aperture; SLM, spatial light modulator; SMT, single-molecule tracking; SPIM, selective plane illumination microscopy; TIRF, total internal reflection fluorescence; TL, tube lens.

Single-molecule detection has changed the way we study complex biological systems (3–7), allowing us to see subpopulations, stochastic processes, or minor reaction pathways embedded in the systems (8–10). Without the need to synchronize the molecular states (70), single-molecule detection enables direct measurement of the association/dissociation kinetics among individual molecules (11, 13, 30, 71, 72). While the early works of single-molecule detection have revealed subspecies in a mixture (73, 74) and protein-folding pathways (75), the use of a flow-through, confocal scheme limits the observation window to about 1 ms (76, 77), thus producing little information on the single molecules. In contrast, TIRF microscopy (Figure 1*a*) allows us to observe surface-tethered (78) or membrane-bound (14) single molecules for an extended period [up to tens of minutes (26), only limited by photobleaching]. Although critical information on enzyme reactions [e.g., walking of myosin V (34) and rotation of ATP synthase (79)], receptor dimerization dynamics [e.g., G protein–coupled receptor dimerization (13)], and cargo transport mechanisms [e.g., through the nuclear pore complex (19)] has been obtained, observation by TIRF microscopy is limited to the 2D space right above the cover slip. The pseudo-TIRF mode, termed highly inclined and laminated optical sheet (HILO) microscopy (80) (Figure 1*a*), enabled single-molecule observation inside mammalian cells (47, 80). Although important processes such as the searching mechanisms of transcription factors in nucleus were revealed (81), the 7-μm-thick optical sheet of HILO mode did not provide a good signal-to-background (S/B) ratio for high-quality SMT (56, 82).

Different from the TIRF microscopy, light-sheet microscopy (LSM) is a 2D optical sectioning technique that can visualize the whole cell or whole organism (83) (Figure 1*b*,*c*). However, it was not until the creation of a thin light-sheet (≤3 μm), using a high numerical-aperture (NA) objective (56, 84, 85) or Bessel plane illumination (86), that enabled SMT on a light-sheet microscope (Figure 1*d*). The current lattice LSM has the sheet thickness of 400 nm across a 40 × 80 μm field of view (29), which is thin enough to suppress most of the fluorescence background to achieve high-quality SMT (Figure 1*e*).

It is possible to achieve 3D-SMT by rapidly scanning a volume using a lattice light-sheet. One example is the 3D-SMT of transcription factor Sox2 in the whole nucleus, achieving an imaging speed of 50 ms per slice and 3 s per volume (67). In this example, HaloTag-Sox2 molecules were labeled with the membrane-permeable dye JF_549_. After repeating the volumetric scan 500 times, the 3D positions of Sox2 were tracked. Although interesting target search dynamics of Sox2 in the embryonic stem cell nucleus were revealed, this light-sheet scanning method is only suitable to track slow moving molecules in cytosol or nucleus (on the order of 0.1–1 μm^2^/s, such as Sox2 diffusing within the enhancer clusters or the heterochromatins).

The superior optical sectioning capabilities of TIRF, HILO, and advanced LSM enable 2D-SMT. However, without axial scanning, these tools cannot provide information on the molecule’s axial movement. Considering that most intracellular and some membrane-bound motions are inherently 3D, a 3D-SMT technique is highly desired.

## 3. THREE-DIMENSIONAL SINGLE-MOLECULE TRACKING AND IMAGING TECHNIQUES

Three key methods extend 2D wide-field observation into the 3D space and enable 3D tracking of single molecules, including point-spread-function (PSF) engineering, multiple plane imaging (92–95) and interferometry (96, 97). Some of these methods are becoming standard techniques, such as astigmatism-based 3D imaging. They are often implemented at core facilities, and many commercial systems are offered.

### 3.1. Multifocal Plane Microscopy

An early method to achieve 3D-SMT is multifocal plane microscopy (MPM), which can be realized by using one (94), two (92, 93), or four (98) cameras or by phase modulation (95). Because each focal plane has an imaging depth of approximately ±1 μm, four focal planes can cover a range of 8 μm or more in the z direction (98), making MPM suitable for molecular tracking in mammalian cells. While the concept of MPM is straightforward, the spacings between distinct focal planes need to be carefully chosen to yield an appropriate 3D localization accuracy (99). Although biplane imaging can be achieved by using a single EM-CCD (electron-multiplying CCD) (94), MPM with four focal planes typically requires four EM-CCDs, making the system prohibitively expensive (98).

**Figure 2:**
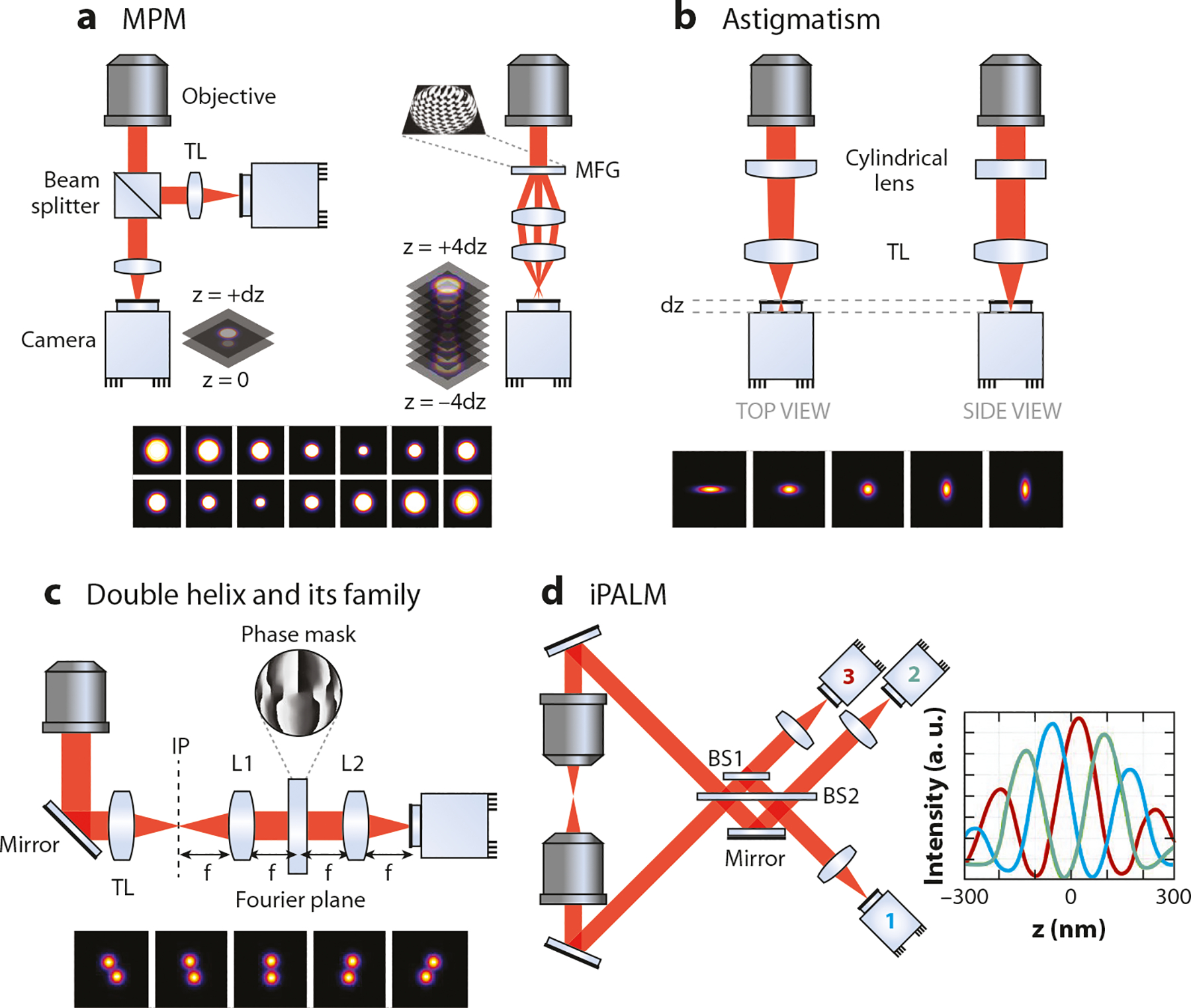
Different 3D-SMT and imaging techniques. (*a*) MPM: (*left*) biplane microscopy (92) and (*right*) 9-plane MPM (95). (*b*) PSF engineering with astigmatism (60, 102, 103). (*c*) PSF engineering using a phase mask in the Fourier plane (31, 104). L1 and L2: two lenses in the 4f system. (*d*) iPALM (96). Abbreviations: 3D, three-dimensional; BS1, 66:33 beam splitter; BS2, 50:50 beam splitter; dz, focus step between successive planes; f, lens focal length; IP, intermediate plane; iPALM, interferometric photoactivation and localization microscope; MFG, multifocus grating; MPM, multifocal plane microscopy; PSF, point-spread-function; TL, tube lens.

Alternatively, by taking advantage of phase modulation, MPM with nine equally spaced focal planes can be realized by using only a single camera (95) (Figure 2*a*). In this scheme, a specially designed diffractive multifocus grating splits and shifts the focus of the sample emission light to form an instant focal series, where each focal plane corresponds to a diffractive order of the multifocus grating (95) and has an interplane spacing of ~440 nm (100). Although MPM enables rapid volumetric imaging of the whole cell without the need for scanning (100), the lack of optical sectioning and splitting of light into multiple planes lead to a reduced S/B ratio and weaker signals, making MPM more suitable to track single molecules heavily labeled with fluorescent proteins (101). However, taking advantage of chemigenetic labeling, Liu’s group (95) tracked single HaloTag-Sox2 molecules labeled with TMR in embryonic stem cells using the 9-plane MPM (Figure 2*a*). Interestingly, they later switched to the aforementioned scanning lattice light-sheet method to track the same transcription factor (67). Although the 3D-SMT data obtained by the 9-plane MPM provided mechanistic insights into the target search process by Sox2, the data by the scanning lattice light-sheet shed light on how the 3D spatial distribution of enhancer sites might affect target search dynamics (67). Lattice LSM clearly has an advantage in tracking molecules in a high-background environment and imaging multicolor species, while the 9-plane MPM achieves a much faster volumetric imaging rate (33 ms versus 3 s per volume).

### 3.2. Point-Spread-Function Engineering

A more popular approach to achieve 3D imaging is to embed the molecule’s axial position information into the 2D image. This can be realized by modifying the PSF of an imaging system by inserting an additional optical component [e.g., a cylindrical lens (102), a deformable mirror (103), or a spatial light modulator (SLM) (104)] into its detection path. After modification, the PSF is no longer symmetrical to the focal plane, and the molecule’s z position can be discerned from the asymmetric PSF with a position uncertainty even smaller than the diffraction limit of light (105).

Astigmatism imaging is the simplest form of PSF engineering (102) for 3D-SMT (60). By placing a weak cylindrical lens [or a deformable mirror (103)] before the tube lens to introduce astigmatism, horizontal rays and vertical rays are focused on two offset focal planes (105) (Figure 2*b*). As a result, PSFs of the fluorescent molecules that reside in the average focal plane (approximately halfway between the horizontal and vertical ray focal planes) have a circular shape, but the shape becomes ellipsoidal when the molecule is above or below the average focal plane. The centroid and the ellipticity of the PSF image are then used to determine the lateral (x and y) and axial (z) positions of the molecule, respectively (106). Because of its simplicity, astigmatism imaging has been incorporated into various types of microscopy, including super-resolution microscopy (106), temporal focusing multiphoton microscopy (107), and LSM (108, 109). By combining astigmatism imaging with stochastic optical reconstruction microscopy (STORM), 3D super-resolution imaging with a lateral resolution of 20 nm and an axial resolution of 50 nm has been achieved (105). However, when a weak cylindrical lens is used, the z-tracking range of an astigmatism PSF is only ±0.4 μm.

To further extend the z-tracking range, PSF has to be engineered through phase modulation of the fluorescence emission, which can be achieved by placing a phase mask at the Fourier plane of a 4f optical system (104) (Figure 2*c*). In the 4f system, the objective and tube lens produce an image of the sample at an intermediate plane. The lens L1 placed at a distance f from this intermediate plane performs Fourier transform of the image at a distance f behind the lens. The transform is then phase modulated by a reflective SLM, reverse Fourier transform is performed by the second lens L2 (at the distance f to the SLM), and the transformed image is projected onto a camera (110). One such example is the double-helix PSF (DH-PSF) (111), which is achieved by placing a specially designed phase mask at the Fourier plane of the 4f system. With DH-PSF, a single emitter’s image is transformed into two lobes, where the midpoint between the two lobes of the DH-PSF denotes the lateral position of the emitter and the angle between the two lobes denotes the z position (Figure 2*c*). DH-PSF not only has a z tracking range larger than that of astigmatism imaging (1 μm versus 0.8 μm), but it also exhibits superior and uniform precision (a smaller Cramér-Rao lower bound) over the tracking range (112). Following the same philosophy, other phase mask designs have been proposed to further extend the z-tracking ranges, creating tetrapod PSF (113), self-bending PSF (114), corkscrew PSF (115), and bisected pupil PSF (116), typically at the cost of a reduced S/B ratio. It should also be noted that any optical systems using phase modulation [including the 9-plane MPM (95)] need to be rigorously calibrated to avoid any artifacts raised by field-dependent variation and chromatic aberration. To further improve the S/B ratio, DH-PSF can be combined with a 2D optical sectioning technique such as LSM for high-quality 3D-SMT (117, 118).

### 3.3. Interferometry

The axial position of the molecule can also be encoded in a 2D image using multiphase interferometry. In the 4Pi microscopy configuration, self-interference of single photons creates an interference pattern along the axial direction (96, 97). Depending on the z-position of the single molecule, the optical path lengths to the three cameras vary, leading to distinctive intensity ratios among the acquired images. At least three images of different phase delays are needed for z-position determination (96) (Figure 2*d*). Whereas amazing details in nanoscale structures of focal adhesions (119), cadherin-based adhesions (119), and cytoskeleton organization in embryonic stem cells (120) were revealed by the interferometric 3D microscopy [coined as iPALM (96)] (Figure 2*d*), its z-tracking range is limited to *λ*/n (31). In addition, 4Pi microscopy requires a perfect alignment condition and a thin sample, preventing the widespread use of this technology. Table 1 summarizes the advantages and disadvantages of several key SMT and SMI techniques.

## 4. THREE-DIMENSIONAL FEEDBACK-CONTROL SINGLE-MOLECULE TRACKING AND IMAGING

Although feedback-control tracking systems often only track one molecule at a time, they have several advantages over the nonfeedback systems. First, with the feedback loop to keep the molecule inside the detection volume (11) or drive the detection volume to follow the molecule path (21) for tracking, the 3D tracking range is no longer limited by the imaging depth or the field of view of the high NA objective (21, 42). Second, unlike the phase modulation methods for 3D-SMT (95, 104), there is no need for complicated PSF calibration to avoid tracking artifacts. Third, by using a femtoliter-sized detection volume, feedback-control systems are particularly suitable for 3D-SMT in a high-background environment (11, 21, 24, 121). Fourth, most feedback systems use single-pixel detectors such as avalanche photodiodes or photon multipliers for tracking. It is thus possible to time stamp the detected photons using a pulsed laser and time-correlated single-photon-counting (TCSPC) module for monitoring the fluorescence lifetime (11, 21) and the antibunching behavior (23, 122) of the tracked molecule. However, other than the low throughput, feedback systems often need to be combined with another imaging modality to gain visual access to the cellular environment where the molecule is tracked (42, 123).

### 4.1. Multidetector Feedback-Control 3D-SMT

**Figure 3:**
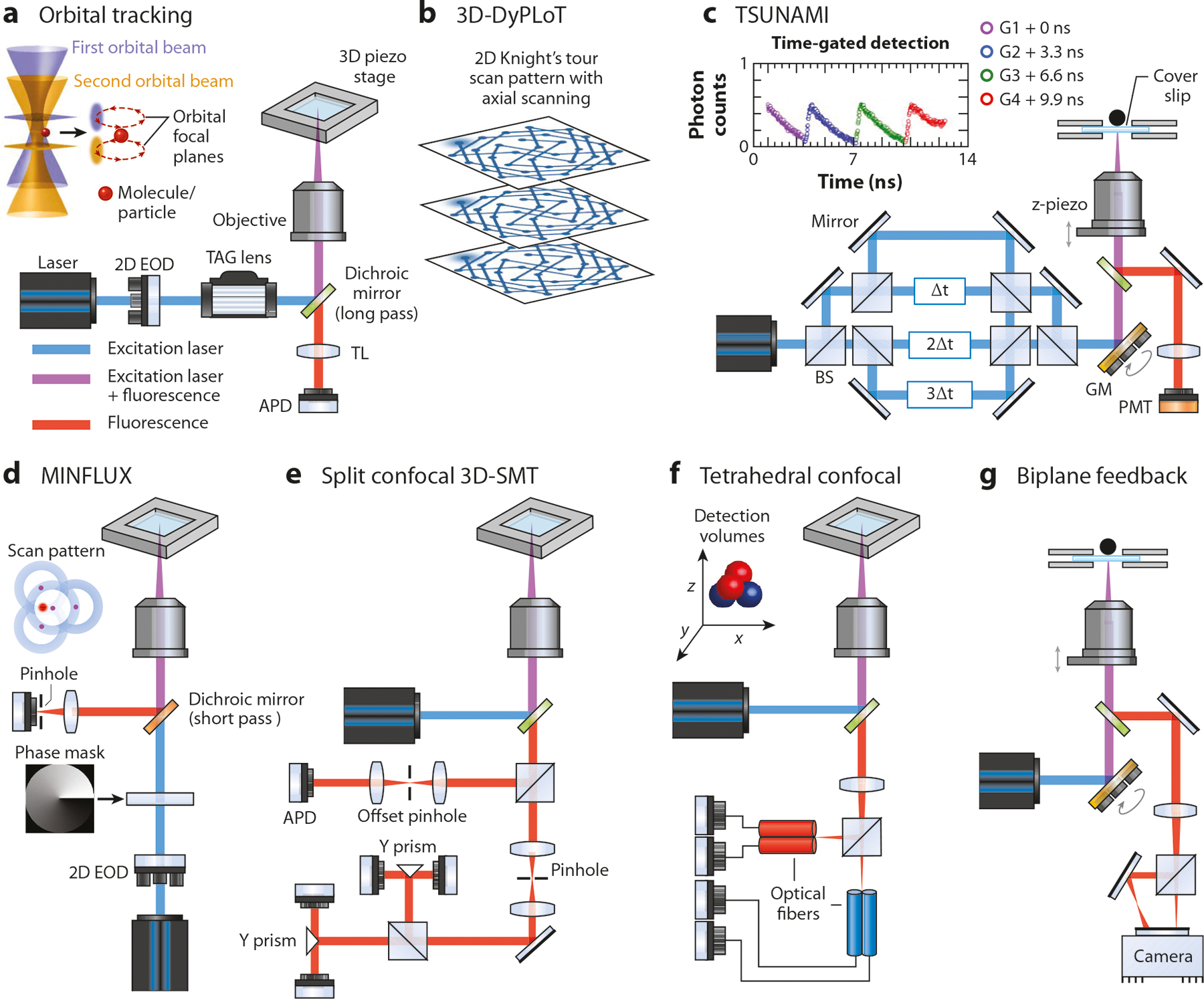
Different tracking modalities developed for 3D-SMT tracking. (*a*) Orbital tracking (129), (*b*) 3D-DyPLoT (132, 133), (*c*) TSUNAMI SMT (21, 136), (*d*) MINFLUX (140–142), and (*e*) split confocal 3D-SMT (125, 126). (*f*) Tetrahedral confocal detection feedback tracking (127, 195). (*g*) Biplane feedback tracking (196). Abbreviations: 2D, two-dimensional; 3D, three-dimensional; APD, avalanche photodiode; BS, beam splitter; EOD, electro-optic deflector; GM, galvo mirror; PMT, photomultiplier tube; SMT, single-molecule tracking; TAG lens, tunable acoustic gradient index of refraction lens; TSUNAMI, tracking of single particles using nonlinear and multiplexed illumination.

Berg (124) first proposed the idea of 3D feedback-control tracking using multiple detectors in 1971 and implemented it for tracking bacteria, where scattered light, instead of fluorescence, was used for tracking. It was not until three decades later that tracking fluorescent nanoparticles or molecules became possible with this multidetector strategy, achieved separately by Yang’s group (42, 125, 126) and Werner’s group (122, 127). Both of their approaches use spatial filtering to improve the S/B ratio and a feedback loop–controlled xyz piezo stage to keep the molecule in the center of detection volumes for 3D tracking. In Yang’s approach, a pinhole is placed at the focus of the tube lens but slightly offset axially (Figure 3*e*). The fluorescence intensity through the pinhole will change as the molecule moves axially, thereby providing the z-position information. To detect the molecule’s lateral position, the fluorescence emission is projected onto the ridges of two orthogonal prism mirrors, which split the signal into the two single-photon detectors. When the molecule is centered, the detectors receive the same number of photons. When the molecule moves laterally, the photon count difference between the detectors (normalized by the total photon count, termed error signals) will vary accordingly (Figure 3*e*). Signals from the five detectors (one for the z-position, two for the x-positions, and two for the y-positions) are fed to the controller, which sends a command to the xyz piezo stage to bring the molecule back to the laser focus center for tracking. By combining confocal tracking with two-photon scanning microscopy, Yang’s group (42) has monitored cellular uptake of peptide-coated nanoparticles with a wide range of spatial and temporal resolutions.

Instead of using five detectors to achieve 3D confocal tracking, Werner’s group (122, 127) used only four (Figure 3*f*). In their approach, the emission is split into two beams, and each beam focuses on the center of a custom-made fiber bundle that consists of two multimode optical fibers. Each fiber serves as a spatial filter for the avalanche photodiode (APD) connected to it. The two fiber bundles are orthogonally orientated and axially offset. The resulting detection volumes form a tetrahedral geometry in the sample space (Figure 3*f*). A fluorescent molecule right in the center of the detection tetrahedron would give equal photon counts in the four detectors, but any displacement from the center would lead to asymmetric photon count distribution. This asymmetry, known as error signal, forms the basis for a feedback loop that drives the xyz piezo stage to reposition the molecule at the center of the detection tetrahedron. Taking advantage of the single-photon detectors, Werner’s group has demonstrated lifetime measurement (122), photon-pair correlation analysis (i.e., antibunching) (121), and time-gated detection (128) (beneficial for SMT in a high-background environment, e.g., inside a cell) together with 3D-SMT, which are not possible with camera-based tracking.

### 4.2. Single-Detector Feedback-Control 3D-SMT

One of the first feedback-control 3D-SMT designs is the orbital tracking. In this scheme, the laser beam is circularly scanned around the emitter [enabled by galvo mirrors (129), acousto-optic modulators (130), or resonant beam deflectors (129)] at the frequency ω_xy_. When the molecule is right at the center of the scanning circle (Figure 3*a*), there is no signal intensity fluctuation during a scanning cycle. However, when the molecule deviates from the center, a sinusoidal variation of the signal is observed over time. Therefore, the molecule’s lateral position can be derived from the magnitude and phase of this sinusoidal fluorescence signal by Fourier transform of the signal (131). To obtain the emitter’s axial position, the focus is shifted between the two axial planes by moving the objective, using an electrically tunable lens or a tunable acoustic index gradient lens. The molecule’s axial position is calculated from the difference of the fluorescence intensity in the two focal planes (Figure 3*a*). Using the real-time 3D emitter position relative to the orbital laser scan, active feedback is achieved by moving the center of the scanning circle to track the emitter’s position. The use of two SPADs (single-photon avalanche diodes) to simultaneously monitor the two offset focal planes bypasses the need of the axial scanning, further improving the temporal resolution. The fluorescence emission is split by a 50:50 beam splitter and focused through two separate confocal channels corresponding to image planes slightly above or below the focus plane of the excitation laser (Figure 3*b*). These methods extract the emitter’s axial information only from intensity measurements, which are subject to low-frequency noise, including fluorescence fluctuation due to the emitter’s dynamics. Such methods have succeeded in tracking only objects that move slowly or lack intrinsic photophysical dynamics. To overcome the limitation, a new method was developed to encode the emitter’s axial position into the frequency component of the fluorescence emission. Two modulated laser beams rotate at the same frequency ω_xy_ and are focused at different depths (separated by ~1 μm) inside the sample. More importantly, the optical powers in the beams are modulated 180° out-of-phase at the frequency ω_z_ by an acousto-optic modulator (130) or acousto-optic deflector (132), thus allowing the molecule’s axial position to be encoded in the ω_z_ frequency component of the fluorescence signal. This tracking method was used to investigate the behavior of nanoparticles at the silicone oil–water interface and found a nonlinear relationship between the diffusion coefficient and the particle size for small particles on the surface (132). The binding of DNA origami and quantum dots was also investigated with this method (133).

An alternative to orbital scanning is the discontinuous scanning method. The method was first implemented with a knight’s tour pattern in the ABEL trap (134). Instead of continuously scanning in a circle, the laser beam is discontinuously scanned across a group of points on a knight’s tour pattern (Figure 3*b*). This heuristic cycles through grid points to sample the entire area, rather than from side to side as in a raster scan. This allows feedback to be applied on every photon detected without the need of waiting for a scan to complete, facilitating the tracking of fast-moving particles that might move across the scan area faster than a raster scan does. Recently, the method was extended to 3D tracking, creating a 3D dynamic photon localization tracking system termed 3D-DyPLoT (132, 133) (Figure 3*b*). This method could further increase the size of the tracking area and allow tracking of faster molecules, at the price of reduced localization precision.

In contrast to the orbital tracking and the 3D knight’s tour tracking, the tetrahedral tracking (121, 122, 127, 128) provides a better S/B ratio because of spatial filtering. Besides, the laser beam is locked directly on the molecule for tracking, rather than having a small offset from the molecule. Furthermore, tetrahedral tracking can achieve a higher temporal resolution because it does not require laser scanning to build up an intensity time trace for position estimation. Tetrahedral tracking can be divided into tetrahedral detection and tetrahedral excitation. Tetrahedral detection techniques typically require 4–5 single-photon counting devices to track single molecules in the 3D space (discussed in Section 4.2). However, tetrahedral excitation methods can be implemented with a single detector. The first tetrahedral excitation-based tracking microscopy was developed using four alternately pulsed laser diodes (135). Four laser diodes were coupled into single-mode fibers and attached to beam collimators with offsets to create two converging and two diverging beams. The four beams were combined by beam splitters and formed four overlapping excitation foci in tetrahedral geometry at the focus of the objective lens. A counter-timer card was used to modulate four laser diodes and count photon arrival pulses from the SPAD. Any xyz displacement of the molecule from the center can be estimated via the normalized photon count difference between the four foci. A closed feedback loop then drives the 3D piezo stage to lock the excitation tetrahedron on the molecule for tracking.

Following the tetrahedral excitation strategy, our group developed a 3D tracking microscope, termed TSUNAMI (tracking of single particles using nonlinear and multiplexed illumination) (21, 136), that only requires one photomultiplier tube to achieve 3D-SMT (Figure 3*c*). The approach is based on passive pulse splitters used for nonlinear microscopy to achieve spatiotemporally multiplexed two-photon excitation and temporally demultiplexed detection to discern the 3D position of the molecule. In TSUNAMI, multiplexed illumination is realized by splitting the pulsed laser from a 76 MHz Ti-sapphire oscillator into four beams, with each beam pulse delayed by 3.3 ns (one-fourth of the laser repetition period) relative to its preceding pulse. These beams are focused through a high NA objective at slightly offset xyz positions, resulting in four two-photon excitation volumes arranged in a tetrahedral geometry. In our case, the four excitation volumes receive laser pulses at different time frames. With TCSPC analysis, each detected photon is assigned to a 3.3-ns-wide time gate (G1–G4 in the fluorescence decay histogram) and thus can be attributed to a specific excitation volume. When the molecule sits at the center of the excitation tetrahedron, the photon counts are approximately equal in all four time gates (Figure 3*c*). Any xyz displacement of the molecule from the center can be estimated via the normalized photon count difference in the four time gates (i.e., error signal). A closed feedback loop then drives the galvo mirrors and the objective z-piezo stage locking the excitation tetrahedron on the molecule for tracking.

A two-photon microscope by nature, TSUNAMI enables multicolor imaging and an imaging depth that the traditional one-photon feedback SMT microscopes cannot achieve. Our group (21) has demonstrated 3D tracking of epidermal growth factor receptor complexes at a depth of ~100 μm in live tumor spheroids. At shallow depth, TSUNAMI has localization accuracy as good as 35 nm and temporal resolution down to 50 μs (with bright fluorophores). Our technique was recently expanded to incorporate two-color detection (25). The multicolor extension of TSUNAMI can simultaneously localize two spectrally distinct targets separated by distances of 33 to 250 nm with 15-nm tracking precision. The technique allows us to measure the 3D position, the 2D rotation, and the separation distance between closely linked targets. We demonstrated the capability of monitoring the landing of the anti-EGFR IgG-conjugated dumbbell-shaped tracers on the plasma membrane and revealed that the interactions between antibody and epidermal growth factor receptors (EGFRs) confined the translational and rotational motions of tracers.

Despite the simplicity in implementation, it is worth noting that the error signal analysis used in the original TSUNAMI and tetrahedral confocal tracking microscopes is not optimal for molecular position estimation. Our recent work (137) demonstrated that a maximum likelihood estimator [initially developed by Hell and Eggeling’s groups for their nonfeedback 2D confocal tracking microscope (138, 139)] could provide a much better axial position estimate without sacrificing lateral localization accuracy or temporal resolution.

Extended from the orbital scanning, MINFLUX utilizes a doughnut-shaped laser beam spot to scan through a small number of selected locations on a circle (140–142). Keeping the particle at the illumination minimum minimizes the total number of photons required for tracking, thus enabling more position information to be obtained within the photon budget (Figure 3*d*). This concept contrasts with the abovementioned methods, where more photons are desired for better localization precision, at the price of reduced track duration due to early photobleaching. While the standard 2D-MINFLUX approach rapidly scans the doughnut-shaped beam spot among the four points (three on a circle and one at the center) (140), the newly developed version, p-MINFLUX (143), sends temporally offset pulses to the four points, similar to our TSUNAMI method. Although this scheme eliminates the need for beam scanning, it requires a pulsed laser for excitation and time-gated electronics for data acquisition and analysis.

Orbital tracking, tetrahedral tracking, and MINFLUX microscopes are superior to camera-based tracking systems in probing the fast dynamics of a single molecule. However, it is equally important to find out how the rapid single-molecule motion fits into the context of the entire biological system. This lack of contextual information (e.g., cellular microdomains or neighboring molecules) can pose the risk of misinterpreting molecular behaviors. To solve this issue, spinning disk microscopy (123) and two-photon laser scanning microscopy (42) have been integrated into feedback-control tracking microscopes to provide a view of the cellular environment where the rapidly diffusing molecules reside.

## 5. ANALYTICAL MEASUREMENTS

Single organic dyes can emit only 10^5^–10^6^ photons before photobleaching. This determines not only how precisely we can localize a molecule but also how long we can track a single dye. If all emitted photons are collected in a single image frame, localization precision (28) can be as good as 1.5 nm (34, 144). However, for a trajectory with track duration of 50 frames, the localization precision of the single molecule in each frame can only be 10 nm, without considering the influence of background fluorescence from the environment (27). To overcome the background fluorescence and achieve a comparable localization precision, SMT in live cells often needs twice as many photons as required for in vitro measurements. When the photon budget remains the same, this means either a 50% reduction in track duration or a 33–50% decrease in single-molecule localization precision in live-cell experiments. As researchers need long trajectories (200–2,000 frames or longer) to elucidate the molecular behaviors, and photobleaching probability typically follows a single exponential decay, an SMT experiment for one particular cellular condition needs to be repeated many times to obtain a sufficient number of long tracks for analysis. This is the key reason that SMT in live cells is much more challenging than that in vitro. What is even more challenging is that the background fluorescence in live cells can fluctuate significantly during the experiment. Using receptor tracking in plasma membranes as an example, the fluctuating background comes from fluorescently labeled vesicles and molecules approaching (or leaving) the receptor of interest. To mitigate the background noise effect, the fluorescently tagged molecules’ concentration is generally kept below 15 nM (27). To differentiate individual receptors on plasma membranes, the expression level of receptors is usually less than 1 copy per μm^2^ (which gives <3,000 fluorescent receptors per cell). From a data acquisition perspective, all observed molecules should be tracked and considered in the trajectory analysis. In this section, we discuss the key methods to extract important dynamic (motional modes), kinetic (association/dissociation rates and equilibrium constant), and clustering (oligomerization) information of the tracked molecules.

### 5.1. Single-Molecule Trajectory Analysis

Extracting useful information from single-molecule trajectory data can be as challenging as performing SMT in live cells (145). Factors such as background fluorescence, detection shot noise, blinking of fluorophore, resolution of the imaging system, and density of the molecules all affect the performance of a connection algorithm to plot the molecular trajectory. For connection algorithms, readers are encouraged to refer to prior reviews (146–148). Here we are not focusing on trajectory generation algorithms, but on trajectory analysis algorithms. Trajectory analysis methods have been continuously evolving over the past two decades, with a number of methods widely used by researchers around the world and having open-source packages available online, such as hidden Markov model (HMM)-based methods (149, 150), Bayesian statistics (151, 152), and machine learning (ML)-based (153, 154) and deep learning (DL)-based (15, 155) techniques. These methods allow researchers to determine the number of diffusive states, the occupancy of each state, and the transition probabilities among these states. Here, we summarize the advantages and disadvantages of commonly used algorithms that have provided insights into the mechanisms of transport in live cells and the role of diffusion-regulated cellular function (Table 2).

#### 5.1.1. Trajectory-relevant physical properties.

**Figure 4:**
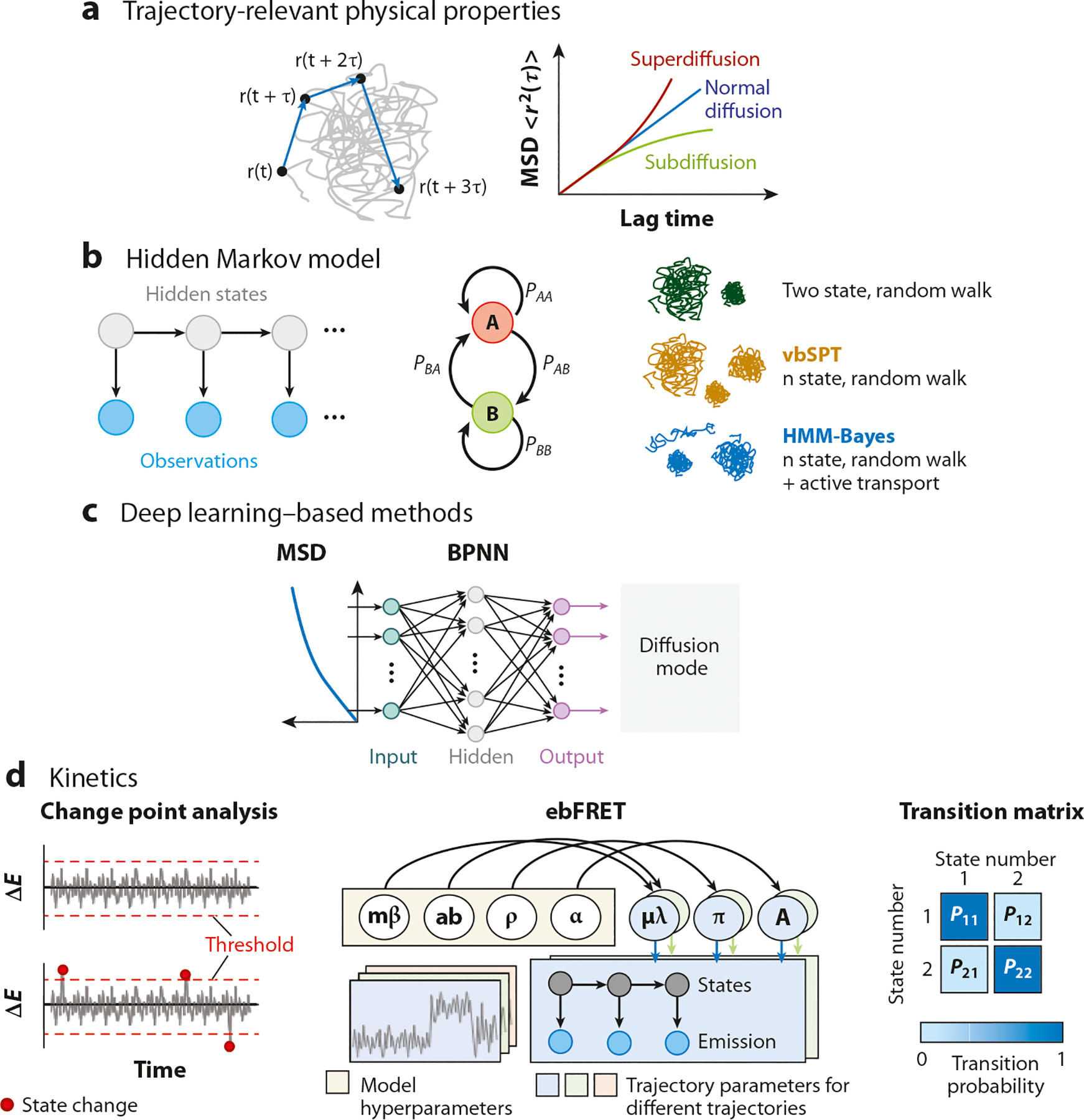
Trajectory analysis methods. (*a*) Trajectory-relevant physical properties (156, 157). Each trajectory yields an MSD curve, which can be used to characterize the diffusion type. (*b*) HMM (149, 161, 162). HMM is a probabilistic model to predict the sequence of hidden states and observations, where each state can have different diffusion coefficients or types. (*c*) Deep learning–based methods (155). The BPNN or convolutional neural network can detect the local and transient diffusion behavior from hundreds of tracking data. (*d*) Algorithm to calculate molecular association/dissociation kinetics (11, 72, 164, 168). Change point analysis and ebFRET applied to the single-molecular trajectory allow characterization of kinetics information of the molecular interaction. Abbreviations: BPNN, back-propagation neural network; ebFRET, empirical Bayesian-based fluorescence resonance energy transfer; HMM, hidden Markov model; MSD, mean-squared displacement; vbSPT, variational Bayes single-particle tracking.

The mean-squared displacement (MSD) analysis is the most straightforward approach widely employed to extract the diffusion coefficient value and identify the type of the diffusion regime of molecules (156, 157). The MSD curve is typically plotted as a function of the lag time. In the case of Brownian diffusion in an isotropic medium, the MSD stays linear, and the underlying diffusion coefficient can be obtained from the slope of the fitted linear model. However, other diffusion types such as anomalous diffusion change the linear dependence into a nonlinear one. The nonlinear least-squares fitting approach is utilized to obtain a parameter, *α*, depicting the nonlinear relationship of MSD with lag time. The trajectory is then categorized as either superdiffusive (*α* > 1) or subdiffusive (*α* < 1) process (Figure 4*a*). However, measurement errors in the real world are inevitable, which degrade the fitting accuracy of an experimental MSD curve. Beyond MSD, the distribution of relative angles of the motion provides supplemental information about the diffusive process. Dinner’s group (158) utilized this parameter to illustrate the common signature of directional motion in the insulin granules and filament motor system, which could not be characterized by the MSD analysis. Recently, Taddei’s group (159) analyzed the diffusion of Rad52 molecules inside the repair foci using a survival probability curve, providing more details of the diffusive behavior of Rad52 around the boundary of subcompartments.

#### 5.1.2. Hidden Markov model and hidden Markov model–based methods.

Although heterogeneous diffusion behaviors of membrane receptors can be observed using MSD analysis (160), the reliance on the averaged measure poses difficulties for characterizing the transient changes in diffusion within a single trajectory. The HMM was introduced to identify the diffusion states and extract the transition probabilities between different states from the single-molecule tracking data (149, 161, 162) (Figure 4*b*). Burroughs’ group (163) further developed the HMM harmonic potential well confinement algorithm to detect the diffusive behavior of the gold nanoparticle-tagged lipids in model membranes. This algorithm can partition trajectories into periods of free diffusion and confinement states. With a clear view of the confinement events, molecular actions such as receptor clustering and hop diffusion become observable.

To avoid the bias in determining the number of diffusive states, Elf’s group (150) employed the variational maximum-evidence approach to develop an analytical tool, termed variational Bayes single-particle tracking (vbSPT) (Figure 4*b*). Because it is unlikely for a complex model to generate data that could be obtained from a simpler model with a smaller parameter space, the merit of the maximum-evidence-based method is to penalize the overly complicated model (164). In contrast to the conventional HMM method, the vbSPT can learn the parameters and make the model selection based on the number of hidden states. As many interactions of the single molecules in the intracellular environment are still unknown even using the purified samples in the test tube, the vbSPT method opens the door to probing and studying these transient or rare interactions. However, existing Bayesian HMM is restricted to modeling purely diffusive motion. Bathe’s group (165) applied the Bayesian model selection to Bayesian model selection to infer transient transport states from trajectories of mRNA-protein complexes in live mouse hippocampal neurons (Figure 4*b*).

#### 5.1.3. Bayesian statistical methods.

An alternative to MSD- and HMM-based approaches for discriminating disparate complex diffusion modes is the Bayesian statistical method (166). This method utilizes Bayes’ theorem to update the knowledge about the parameters with the information encoded in the observations. Leake’s group (151) presented the Bayesian ranking of diffusion process to determine the diffusive types among anomalous, Brownian, confined, and directed diffusion from the protein tracking data. The top-ranked diffusion model selected based on the posterior indicates a significant proportion of the single-molecule trajectories exhibiting confined diffusion. In addition, as the protein motion is also often influenced by the binding energies with its interacting partners, Dahan’s group (152) provided a Bayesian inference framework to map the diffusion and energy landscapes in the cellular surface and investigate the underlying biochemical interactions.

#### 5.1.4. Machine learning– and deep learning–based methods.

Recently, ML and DL methods are becoming more popular in single-molecule image and trajectory classification. Although the ML- and DL-based methods require additional tasks such as data preparation and model training for implementation, these approaches can achieve high classification accuracy. ML enables the identification of motion patterns from the SMT data and allows for continuous improvement of motion classification with the growing amount of data. Sbalzarini’s group (153) implemented the support vector machine to identify different types of human adenovirus motion in host cells using trajectory-derived features. Wiemann’s group (154) utilized the ensemble-based random forest classifier to successfully classify the motion types of the particles inside and outside of lung fibroblasts. Other than the ML methods, DL was also used to analyze single-molecule trajectories (Figure 4*c*). Milhiet’s group (155) applied the back-propagation neural network to detect the local and transient diffusion behaviors of membrane proteins, eliminating the need for a minimum number of observed particle displacements within the trajectory to infer the presence of multiple motion states. Szwabiński’s group (167) demonstrated that the convolutional neural network could be superior to the feature-based methods. Most recently, our group (15) presented a novel 14-layer variant of residual neural network to analyze the trajectory data of a transmembrane receptor and achieved 83% accuracy in breast cancer cell line classification. Although both ML- and DL-based methods provide promising results, further exploitations are indispensable to address the needs of selecting optimal features and speeding up the training without compromising the performance.

#### 5.1.5. Extension from dynamics to kinetics.

Another important piece of information that can be derived from single-molecule trajectories is the kinetics of molecular interactions (11, 72, 164, 168). FRET is the most commonly used method to assess the binding kinetics between the donor and the acceptor. The computational tools developed to probe the conformational dynamics of a surface-tethered molecule can also be used to probe the donor/acceptor fluorescence fluctuation time trace along a molecular trajectory, unveiling the change points (Figure 4*d*). Although the change point analysis is straightforward when the threshold and number of states are predetermined, user biases may deteriorate the change point estimates (169). The step transition and state identification algorithm introduced by Landes’ group (170) reduced user bias by determining the state transition and the optimum number of states via the student’s *t*-test and the minimum description length principle, respectively. In addition, Ha’s group (171) established the HaMMy package that implements HMM to uncover the state evolution. Wiggins’ group (172) developed the variational Bayesian-based tool, termed vbFRET, that enables automatic model selection. vbFRET is similar to vbSPT but has different target properties. The main shortcoming of vbFRET was that it can only model an individual trajectory or multiple trajectories with the same parameters. Accordingly, Gonzalez’s group (173) presented an empirical Bayesian-based approach, ebFRET, to infer features of the prior distribution. This allowed for an accurate analysis of the short time traces with a very low S/B ratio (Figure 4*d*).

### 5.2. Single-Molecule Clustering Analysis

Cell signaling is often triggered by molecular clustering (16). Transmembrane receptors frequently form homodimers or heterodimers upon stimulation (13, 20, 174). Studying molecular cluster structure and organization is important to understand their functions in the cell. Biophysical parameters of the molecular clusters, such as cluster density, cluster size, and cluster distribution, directly associate with a cell’s physiological state. The molecular tracking and imaging methods described in this review also generate single-molecule localization (SML) data that can be used to explore the cluster structure and organization, thus shedding light on the cluster’s function. Here, we discuss the pros and cons of the commonly used clustering analysis methods (Table 3).

In cluster analysis, positions of single molecules are grouped into coherent structures for visualization and interpretation of the clusters. A simple approach for clustering analysis is the Ripley’s K-function that measures the density of molecules in an area as a function of the radius around each molecule in the data set and compares the result to a group of randomly distributed molecules of the same density (175–177). The L-function is a normalization of the K-function (178), which can be used to derive the sizes of the clusters (179). Normalizing the L-function further produces the H-function, whose value fluctuates around zero for uniformly distributed molecules, above zero for clustered molecules, and below zero for dispersed molecules. Although Ripley’s functions do not require any input of initial parameters, they can only quantify the level of molecular clustering globally, not the sizes of individual clusters (16, 33, 180).

In contrast to Ripley’s functions, density-based clustering methods require initial parameters but can define individual clusters (181). Density-based spatial clustering of applications with noise (DBSCAN) is one of the most popular density-based methods, which can identify clusters of arbitrary shapes (180, 181). This method requires two user-defined parameters: a neighborhood radius (ε) and a minimum number of molecules in the neighborhood (MinPts) to initiate the clustering analysis. As the analysis has a minimum threshold for each detection radius, noisy events in the SML data can be filtered out when appropriate parameters are used. Based on these criteria, each localization point is labeled as a core point, an edge point, or a noise point. Core and edge points belong to clusters, while noise points do not. Although this algorithm can assign molecules into individual clusters, the main disadvantage is that the user-defined parameters ε and MinPts strongly affect the clustering analysis outcome (182). In practice, optimizing ε and MinPts is a try-and-error process that requires users to run DBSCAN multiple times (183).

Alternatively, clustering analysis can be performed using Voronoi polygons (184, 185). Voronoi tessellation is generated by dividing the space into regions based on Euclidean distances of one molecule to other neighboring molecules, producing a Voronoi diagram (184). Clusters are defined by connecting adjacent Voronoi cells with similar geometric properties (e.g., size and shape). Voronoi tessellation-based methods usually rely on Voronoi cell size to segment molecular clusters, where the Voronoi cell size is inversely proportional to the molecular density (16). By comparing the Voronoi cell sizes with a reference distribution chosen to be either spatially uniform (184) or randomly distributed (185), the threshold for segmentation of the Voronoi diagram can be determined.

In contrast to the density-based and Voronoi tessellation-based methods, the Bayesian approach is a parameter-free and model-based method (186). SML data are analyzed by several clustering models generated by a statistic method such as Ripley’s K-function. Whereas user bias can be minimized in the Bayesian method, the performance is highly dependent on the model selection.

## 6. CONCLUSION AND OUTLOOK

**Figure 5:**
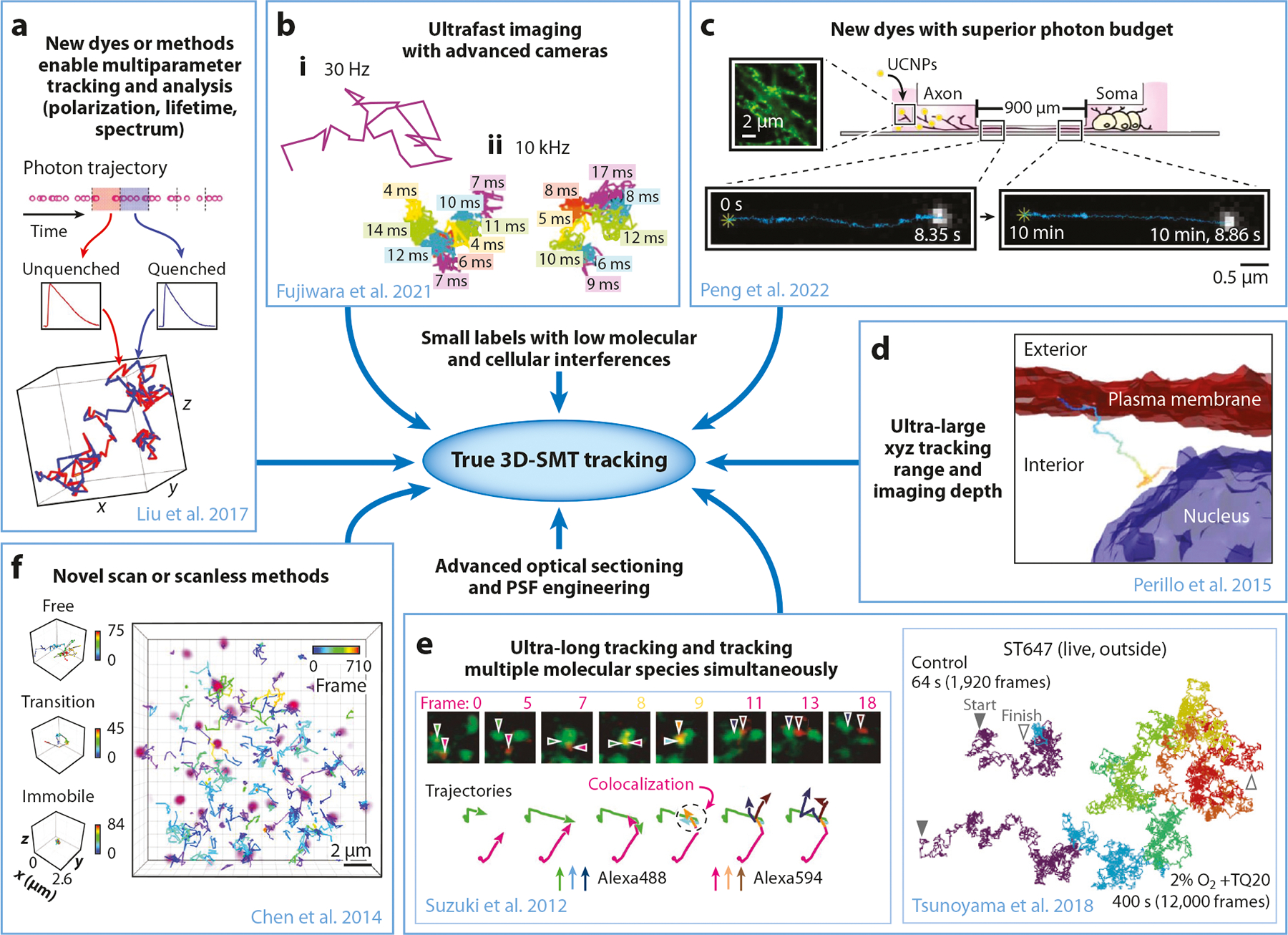
Roadmap to true 3D-SMT in live cells. (*a*) Spatiotemporally resolved single DNA annealing-melting kinetics measurement with fluorescence lifetime. In our DNA model system, the longer lifetime (from unquenched dye) represents the single-stranded DNA state (*red segments*) while the shorter lifetime (from quenched dye) indicates the double-stranded DNA state (*blue segments*). The acquired single-molecule lifetime trace is mapped onto the molecule’s 3D trajectory, providing the temporal and spatial information of annealing-melting events that take place along this 1,015-ms trajectory. Panel adapted with permission from Reference 23; copyright 2017 Royal Society of Chemistry. (*b*) Typical single-molecule trajectories of Cy3-DOPE in intact apical plasma membranes recorded at (*i*) normal video rate and (*ii*) enhanced rates with advanced cameras. Panel adapted from Reference 41 with permission from the authors. (*c*) UCNPs enabled superlong tracking of individual cargos transported by dynein motors in live neurons. Panel adapted with permission from Reference 188 with permission from the authors. (*d*) 3D tracking of epidermal growth factor receptor complexes at a depth of ~100 μm in live tumor spheroids. This example trajectory shows slow diffusive transport in cytosol and interaction with the nucleus. Isocontours of the zoomed-in image stack taken 90 μm deep in a spheroid with plasma membrane (*red*) and nucleus (*blue*) are overlaid with the trajectory (*rainbow path*). Panel adapted with permission from Reference 21; copyright 2015 Springer Nature. (*e, right*) Typical trajectories for ST647 molecules linked to CD47 in the live cell plasma membrane that could be tracked without photoblinking and photobleaching for periods longer than 400 s. (*e, left*) Typical two-color, single-molecule image sequences. CD59 and DAF molecules, labeled with Alexa488-Fab–CD59 (*green*) and Alexa594-Fab–DAF (*red*), respectively. The colocalization of two spots with different colors was determined by measuring the distances between the two determined coordinates. Panel adapted with permission from Reference 26, copyright 2018 Springer Nature; and Reference 38, copyright 2012 Springer Nature. (*f*) Volume rendering of 3D Sox2 single-molecule image (*purple*) superimposed with single-molecule trajectories generated by simultaneous multifocal plane microscopy. Three molecules with distinct behaviors were selectively displayed on the right (*from top to bottom*: freely diffusing particle, particle undergoing a free/bound transition, and immobile molecule). Color bar shows the corresponding frame number. Panel adapted with permission from Reference 86; copyright 2014 Elsevier. Abbreviations: 3D, three-dimensional; PSF, point-spread-function; SMT, single-molecule tracking; UCNP, upconversion nanoparticle.

Future textbooks in molecular, cellular, and developmental biology will not only have beautiful models and schematics that describe molecular interactions, complex formations, transport processes, and signaling pathways, but they will also include videos that show single molecules in action and provide information on timescales, rate constants, equilibrium constants, delivery routes, motion speeds, and oligomer sizes of various molecular machines (27). To realize this dream, a true 3D-SMT method is required, which enables the tracking of a large number of molecules freely diffusing in the intracellular 3D space altogether with an unlimited z-tracking range and spatiotemporal resolution. Besides, such a method should also have a multiplexed detection capability, allowing for the simultaneous observation of a variety of molecular species. Moreover, labels on the molecules of interest are stably bright and will not interfere with any native functions of the molecule. Apparently, there is no true 3D-SMT method today (Figure 5). Using an ultrafast camera system, a single Cy3 dye (which has the lowest tendency to reach emission saturation) can be tracked at 10 kHz or every 100 μs, with 20-nm single localization precision and in a frame size of 14 μm × 14 μm (256 × 256 pixels) (41). While the camera system can be operated at a faster speed, the emission rate of a single organic dye cannot keep up with the camera rate [limited by the triplet bottleneck saturation (41, 187)]. The camera tracking frame rate of 10^4^–10^5^ Hz is likely to be the ultimate rate achievable by the currently available organic dyes (41, 187). New fluorescent labels that bypass the limitations of emission rate bottleneck and photobleaching, such as upconversion nanoparticles, are under development. However, although upconversion nanoparticles have enabled superlong tracking of individual cargos transported by dynein motors in live neurons (188), how to further reduce their sizes and allow for intracellular labeling is still a problem to overcome.

All SMT techniques mentioned in this review have their advantages and disadvantages. Although microscopists continue to push for deeper tracking of multiple molecular species with higher spatiotemporal resolution in three-dimensional tissue samples, another trend is to gain more information from the detected photons, such as polarization (189), fluorescence lifetime (11), and emission spectrum (190) for understanding the state of the molecule (11) or the environment (189) surrounding the molecule. In recent years, hyperspectral imaging techniques combined with fluorescence lifetime measurements have facilitated differentiation of fluorophores, creating methods such as phasor-sFLIM (spectral fluorescence lifetime imaging microscopy) (191). Using a 32-channel PMT detector array and a digital-frequency-domain electronics setup for lifetime acquisition and processing, phasor-sFLIM provides parallel, simultaneous lifetime and spectral detection at each pixel, enabling unmixing of three unknown fluorophores at each excitation wave-length. It is possible to integrate phasor-sFLIM with an SMT technique to create a whole new method that can monitor the change of lifetime and emission spectrum of a single molecule along its trajectory. Such changes on fluorescence signatures can shed light on molecules’ interactions with their environments or other signaling molecules. Other than multiparameter fluorescence detection, scanless imaging methods are under rapid development for high-speed imaging (192–194), which may soon give us a high-speed and high-quality volumetric imaging tool. But of course, little information can be derived from the SMT data if the photon budget is still very limited. We anticipate in the foreseeable future that SMT techniques will continue to evolve and the bottlenecks for the emission rate and photon budget will be lifted. We will then be one step closer to the true 3D-SMT in live cells, tissues, or organisms.

## Figures and Tables

**Table 1:** Overview of SMT or SMI techniques

Category	System	Key features	Advantages	Disadvantages
Single-molecule detection in solution	Confocal microscopy (73, 74)	Femtoliter-sized detection volume achieved by a high NA objective and a pinhole	Standard optical system and simple protocol Ideal for quantification of single molecules	Short observation window (typically 1 ms or less) giving little information on single molecules
2D-SMT near the surface of a cover slip or in a confined space	TIRF (14, 37)	Thin excitation volume generated by evanescent field above the cover slip	Excellent S/B ratio in SMT High-throughput tracking of single molecules	Only observe surface-tethered or membrane-bound molecules Limited temporal resolution (video rate) No information on the axial movement
	HILO (80)	A highly inclined and thin beam produced by a pseudo-TIRF mode	Enables single-molecule detection inside cytosol or nucleus of a live cell	Much higher background compared to TIRF
3D-SMT via PSF engineering	Astigmatism (60, 102, 103)	Axial position information of the molecule encoded in PSF	Excellent z spatial resolution Simple optical setup	Z-tracking range limited to 0.8 μm
	DH-PSF (104) and its family (31)		Z-tracking range up to 20 μm Uniform precision over z-tracking range	Reduced S/B ratio with enlarged PSF Requires complicated calibration
3D-SMT via multifocal plane microscopy	Biplane (92)	Simultaneous image multiple focal planes in the sample space	Simple optical setup	S/B ratio and z spatial resolution are not as good as scanning light-sheet microscopy
	9-plane MPM (68, 86, 95)		Axial imaging range from 2 μm up to 18 μm	
3D-SMT via scanning light-sheet microscopy	Bessel-beam light-sheet microscopy (86) Lattice-beam light-sheet microscopy (67)	Scanning of Bessel or lattice-beam light-sheet in z direction	Excellent z spatial resolution as high as 9.8 nm	Temporal resolution limited by scanning speed
3D-SMT via interferometry	iPALM (96)	Self-interference of emission creating distinct interference patterns in 3 cameras	Excellent z spatial resolution as high as sub-20 nm	Limited z-tracking range Only good for thin samples Temporal resolution limited by camera frame rate
Multidetector feedback-control 3D-SMT	Split confocal (42)	Spatial position information is detected by offset pinholes	Excellent spatiotemporal resolution Acquires fluorescence dynamics while tracking a molecule (e.g., decay histogram)	Requires multiple detectors
	Tetrahedral confocal (23, 122)	Employs a confocal scheme to create four detection volumes in a tetrahedral arrangement around the emitter		
Single-detector feedback-control 3D-SMT	Orbital tracking (197–200)	Scans the beam in a circular pattern around an emitter	Compatible with two-photon excitation scheme	Temporal resolution limited by scanning speed
	TSUNAMI (17, 21, 137)	Scans the beam in a tetrahedral geometry using passive components	Imaging depth up to 200 μm Compatible with multicolor detection Acquires fluorescence dynamics while tracking a molecule (e.g., decay histogram)	Requires a TCSPC system for time-gated analysis of the arrived photons
	3D-DyPLoT (201–203)	Scans the beam in a 3D knight’s tour pattern around an emitter	Enables a larger tracking area Allows for tracking fast-moving particles	Requires fast scanning hardware Reduced S/B ratio due to a large scanning area
	MINFLUX (140–142)	Scans the doughnut-shaped laser beam around an emitter	Reduced photobleaching	Requiring a doughnut-shaped beam and fast scanning hardware

Abbreviations: 2D, two-dimensional; 3D, three-dimensional; DH-PSF, double-helix point-spread-function; HILO, highly inclined and laminated optical sheet; iPALM, interferometric photoactivation and localization microscope; MPM, multifocal plane microscopy; NA, numerical aperture; S/B, signal-to-background; SMI, single-molecule imaging; SMT, single-molecule tracking; TCSPC, time-correlated single-photon-counting; TIRF, total internal reflection fluorescence; TSUNAMI, tracking of single particles using nonlinear and multiplexed illumination.

**Table 2:** Summary of the commonly used methods for trajectory analysis

Category	Method	Input data	Output data	Advantages	Disadvantages
Trajectory-relevant physical properties	Mean-squared displacement (MSD) (156, 157)	Tracking trajectories	Diffusion coefficients Anomalous exponents	Easy to implement	Prone to measurement errors Unable to resolve the short-lived motion
	Distribution of directional change (158)		Relative angles to the reference	Providing supplemental information about diffusive behaviors	Unable to resolve the short-lived motions
	Survival probability (159)		Mean dwelling (residence) time	Providing supplemental information about diffusive behaviors	Unable to resolve the short-lived motions
Hidden Markov model (HMM)–based method	HMM (149)	Tracking trajectories	State paths Transition probabilities	Providing supplemental information on transient motional modes	Limited to Brownian motion Subjective selection of the number of states
	Variational Bayes single-particle tracking (vbSPT) (150)		State paths Transition probability Optimal number of states	Providing supplemental information on transient motional modes Automatic selection of the number of states	Limited to Brownian motion
Bayesian statistical method	Bayesian ranking of diffusion (BRAD) (151)	Tracking trajectories	Diffusion modes	High classification accuracy on motion types	Requiring prior knowledge of motion types
	Bayesian inference (152)		Diffusion coefficients	Providing supplemental information on diffusive behaviors	Requiring prior knowledge of motion types
Machine learning (ML)	Support vector machine (SVM) (153)	Tracking trajectories	Diffusion modes Segmented trajectory	High classification accuracy on motion types	Requiring a feature selection Longer processing time
	Random forest classifier (RFC) (154)		Diffusion modes Segmented trajectories	High classification accuracy on motion types Software already incorporated in ImageJ	Requiring feature selection Longer processing time
Deep learning (DL)	Back-propagation neural network (BPNN) (155)	MSD curves	Diffusion modes	No need for a feature selection High classification accuracy on motion type	Limited diffusion modes available for analysis Need of a training data set Longer processing time
	Residual neural network (ResNet) (15)	Tracking trajectories	Trajectory classification for cell type differentiation	No need for a feature selection Enabling cell type prediction	Need of a training data set Longer processing time
Extension from dynamics to kinetics	Change point analysis (CPA) (169)	Intensity-/lifetime-based traces	Kinetic rate constants	Easy to implement Less computational time	Susceptible to user bias Possibility to generate the wrong number of states Prone to overlooking the events of short-dwell times
	Step transition and state identification (STaSI) (170)		Kinetic rate constants	Less prone to user bias Estimates the number of states best	Requiring multiple steps for implementation Prone to the events of short-dwell times Not for transition detection
	HaMMy (171)		State paths Transition probabilities Kinetic rate constants	Less prone to user bias Restoring fast rate coefficients	Only analyzing a single trajectory or multiple trajectories with the same parameters
	Empirical Bayesian fluorescence resonance energy transfer (ebFRET) (173)		State paths Transition probabilities Optimal number of states Kinetic rate constants	Automatic selection of the number of states Ideal for short trajectory analysis	Undetermined statistical errors Possibility to overestimate the number of states Highly computationally intensive

**Table 3:** Summary of the commonly used methods for cluster analysis

Method	Output data	Advantages	Disadvantages
Ripley’s K-function (175, 178)	H-function, which represents the extent of clustering	Simple protocol and rapid analysis	Limited to homogeneous clusters Has only a single-value output that represents the extent of clustering
DBSCAN (181)	Cluster size and cluster density	Includes a noise removal algorithm Applicable to most structures	Limited to homogeneous clusters Prone to user bias in selecting parameters
Voronoi tessellation (184, 185)	Well-defined cluster region and cluster distribution	Simple protocol and rapid analysis Includes a noise removal algorithm Applicable to filament-like structures	Not compatible with hollow clusters and anisotropic cluster distribution
Bayesian cluster identification (186)	Cluster size and relative density	Includes a noise removal algorithm Not dependent on parameters Not sensitive to changes in localization precisions	Highly computationally intensive
